# Supplementary material for: HIVprotI: an integrated web based platform for prediction and design of HIV proteins inhibitors
Source: J Cheminform. 2018 Mar 9;10:12. doi: 10.1186/s13321-018-0266-y (PMC5845081; doi:10.1186/s13321-018-0266-y)
Supplement: Supplementary file 2 — Additional file 2. Source code of HIVProtI web server. [file 13321_2018_266_MOESM2_ESM.zip › HIVprotI_Source-code/hivprotI/jsme/JME_to_JSME.html]

JME to JSME migration. Part 1


# JME to JSME migration

You have to enable JavaScript in your browser to use JSME !

The variable name "jme" refers to the applet document.jme .
  
The applet can also be accessed via document.jsapplets[0].
  

---

JSME in depict mode:

You have to enable JavaScript on your machine !

You have to enable JavaScript in your browser to use JSME !
